# SARS-CoV-2 Outbreak Investigation Using Contact Tracing and Whole-Genome Sequencing in an Ontario Tertiary Care Hospital

**DOI:** 10.1128/spectrum.01900-22

**Published:** 2023-04-24

**Authors:** Kara K. Tsang, Shehryar Ahmad, Alanoud Aljarbou, Mohammed Al Salem, Sheridan J. C. Baker, Emily M. Panousis, Hooman Derakhshani, Laura Rossi, Jalees A. Nasir, David C. Bulir, Michael G. Surette, Robyn S. Lee, Fiona Smaill, Dominik Mertz, Andrew G. McArthur, Sarah Khan

**Affiliations:** a Department of Infection Biology, London School of Hygiene and Tropical Medicine, London, United Kingdom; b Temerty Faculty of Medicine, University of Toronto, Toronto, Ontario, Canada; c Department of Pediatrics, College of Medicine, Imam Mohammad Ibn Saud Islamic University, Riyadh, Saudi Arabia; d Department of Pediatrics, McMaster University, Hamilton, Ontario, Canada; e Michael G. DeGroote Institute for Infectious Disease Research, McMaster University, Hamilton, Ontario, Canada; f Department of Biochemistry and Biomedical Sciences, McMaster University, Hamilton, Ontario, Canada; g Department of Animal Science, University of Manitoba, Winnipeg, Manitoba, Canada; h Farncombe Family Digestive Health Research Institute, McMaster University, Hamilton, Ontario, Canada; i Department of Medicine, McMaster University, Hamilton, Ontario, Canada; j Department of Pathology and Molecular Medicine, McMaster University, Hamilton, Ontario, Canada; k Epidemiology Division, Dalla Lana School of Public Health, University of Toronto, Toronto, Ontario, Canada; Emory University School of Medicine

**Keywords:** COVID-19, DNA sequencing, outbreak, SARS-CoV-2, Canada, contact tracing, genomics, hospital

## Abstract

Genomic epidemiology can facilitate an understanding of evolutionary history and transmission dynamics of a severe acute respiratory syndrome coronavirus 2 (SARS-CoV-2) outbreak. We used next-generation sequencing techniques to study SARS-CoV-2 genomes isolated from patients and health care workers (HCWs) across five wards of a Canadian hospital with an ongoing SARS-CoV-2 outbreak. Using traditional contact tracing methods, we show transmission events between patients and HCWs, which were also supported by the SARS-CoV-2 lineage assignments. The outbreak predominantly involved SARS-CoV-2 B.1.564.1 across all five wards, but we also show evidence of community introductions of lineages B.1, B.1.1.32, and B.1.231, falsely assumed to be outbreak related. Altogether, our study exemplifies the value of using contact tracing in combination with genomic epidemiology to understand the transmission dynamics and genetic underpinnings of a SARS-CoV-2 outbreak.

**IMPORTANCE** Our manuscript describes a SARS-CoV-2 outbreak investigation in an Ontario tertiary care hospital. We use traditional contract tracing paired with whole-genome sequencing to facilitate an understanding of the evolutionary history and transmission dynamics of this SARS-CoV-2 outbreak in a clinical setting. These advancements have enabled the incorporation of phylogenetics and genomic epidemiology into the understanding of clinical outbreaks. We show that genomic epidemiology can help to explore the genetic evolution of a pathogen in real time, enabling the identification of the index case and helping understand its transmission dynamics to develop better strategies to prevent future spread of SARS-CoV-2 in congregate, clinical settings such as hospitals.

## INTRODUCTION

Hospital outbreaks of severe acute respiratory syndrome coronavirus 2 (SARS-CoV-2) have continued to threaten health care systems and public health since February 2020. Infection prevention and control (IPAC) clinicians rely on the gold-standard molecular diagnosis of SARS-CoV-2 using reverse transcription-quantitative PCR (RT-qPCR) (1) for outbreak detection and surveillance. Accurate diagnostics combined with contact tracing allow for SARS-CoV-2 outbreak management. With the recent paradigm shift toward next-generation sequencing tools, IPAC clinicians are now also combining whole-genome sequencing with traditional SARS-CoV-2 outbreak management practices (2–6). These advancements have enabled the incorporation of phylogenetics and genomic epidemiology into the understanding of an outbreak. Genomic epidemiology can help to explore the genetic evolution of a pathogen in real time, enabling the identification of the index case and helping understand its transmission dynamics to develop better strategies to prevent future spread of SARS-CoV-2 in congregate settings such as hospitals.

The aim of our study was to combine diagnostics, contact tracing, and whole-genome sequencing to understand a SARS-CoV-2 outbreak in a tertiary care center in Ontario, Canada. Using contact tracing and genomic epidemiology, we show evidence of SARS-CoV-2 transmission between patients and health care workers (HCWs) across five wards in the hospital, in addition to a few independent community importations. Using phylogenetics, we exemplify the dynamic nature of SARS-CoV-2 evolution and interpret the outbreak within the Canadian SARS-CoV-2 epidemiologic landscape.

## RESULTS

### Patient characteristics.

A total of 106 cases of SARS-CoV-2 infection were identified, of which 58 were patients and 48 were HCWs. Characteristics of patients are summarized in Table 1. The average age of all patients was 78.9 years old (standard deviation [SD], 12.4 years), and 51.7% (*n* = 30) were male. The case fatality rate among patients was 17% (*n* = 9/58). Most of the cases were attributed to ward A (39.7%, *n* = 23), followed by ward C (32.8%, *n* = 19), ward D (15.5%, *n* = 9), ward E (6.9%, *n* = 4), and ward B (5.2%, *n* = 3). Some patients moved to different rooms during their stay for a variety of reasons, e.g., cohorting COVID-positive patients. Most patients and HCWs were likely unvaccinated, as the hospital outbreak occurred when the COVID-19 vaccination program was only implemented for individuals at long-term care facilities and not yet for HCWs or the general population.

**TABLE 1:** Descriptive characteristics of COVID-positive patient cases in the outbreak^*a*^

Age (yrs)	Sex	Ward	Room no.	Date positive (day-mo-yr)	Nosocomial	SARS-CoV-2 variant
74	F	A	2	3-Dec-20	Confirmed	B.1.564.1
93	F	A	1	3-Dec-20	Confirmed	B.1.564.1
90	M	A	11	3-Dec-20	Confirmed	B.1.564.1
61	M	A	2	3-Dec-20	Confirmed	B.1.564.1
68	F	B	212	3-Dec-20	Confirmed	B.1.564.1
82	M	B	218	3-Dec-20	Confirmed	B.1.564.1
89	M	A	6	4-Dec-20	Confirmed	B.1.564.1
62	F	B	219	4-Dec-20	Confirmed	B.1.564.1
90	M	C	12	4-Dec-20	Confirmed	B.1.1.32
84	M	C	17	6-Dec-20	Confirmed	B.1
87	M	C	17	6-Dec-20	Confirmed	B.1.564.1
87	F	C	17	6-Dec-20	Confirmed	B.1.564.1
64	F	A	7	7-Dec-20	Confirmed	B.1.564.1
92	F	A	3	7-Dec-20	Confirmed	B.1.564.1
70	M	A	9	8-Dec-20	Confirmed	—
66	F	A	12	9-Dec-20	Confirmed	—
94	M	A	2	10-Dec-20	Confirmed	—
69	F	A	1	10-Dec-20	Confirmed	—
92	M	D	14	10-Dec-20	Confirmed	B.1.564.1
78	M	D	2	10-Dec-20	Confirmed	B.1
81	M	E	4	10-Dec-20	Confirmed	—
92	M	E	4	10-Dec-20	Confirmed	B.1.1.231
77	M	D	17	11-Dec-20	Confirmed	B.1.564.1
71	M	D	17	11-Dec-20	Confirmed	—
81	F	C	3	13-Dec-20	Confirmed	B.1.564.1
68	F	C	12	13-Dec-20	Confirmed	B.1.564.1
99	F	C	12	13-Dec-20	Confirmed	—
70	F	C	14	13-Dec-20	Confirmed	B.1.564.1
94	F	C	15	13-Dec-20	Confirmed	B.1.564.1
63	F	A	6	14-Dec-20	Nonnosocomial	—
88	M	D	(3)17	16-Dec-20	Confirmed	B.1.564.1
75	M	A	14	17-Dec-20	Confirmed	—
79	F	A	7 or 17	17-Dec-20	Confirmed	—
92	M	C	18	17-Dec-20	Confirmed	B.1.564.1
53	F	C	5	17-Dec-20	Confirmed	—
78	F	D	1	17-Dec-20	Confirmed	B.1.564.1
48	F	D	(3)17	17-Dec-20	Confirmed	B.1.564.1
68	M	D	1	17-Dec-20	Confirmed	B.1.564.1
87	M	C	3	19-Dec-20	Confirmed	B.1.564.1
88	F	E	2	19-Dec-20	Confirmed	B.1.564.1
91	M	C	5	20-Dec-20	Confirmed	B.1.564.1
87	M	E	3	20-Dec-20	Confirmed	B.1.564.1
53	M	A	11	21-Dec-20	Confirmed	—
75	F	A	9	22-Dec-20	Confirmed	B.1.564.1
86	F	A	14	22-Dec-20	Confirmed	B.1.564.1
67	M	D	2	22-Dec-20	Confirmed	—
86	F	C	16	23-Dec-20	Confirmed	—
52	M	C	2	23-Dec-20	Confirmed	—
90	F	A	12	26-Dec-20	Nonnosocomial	—
71	M	C	16	26-Dec-20	Confirmed	—
92	M	C	4	26-Dec-20	Confirmed	—
70	M	C	8	27-Dec-20	Confirmed	—
79	M	C	5	29-Dec-20	Confirmed	—
90	F	A	15	30-Dec-20	Confirmed	—
77	F	A	6	31-Dec-20	Confirmed	—
92	M	A	6	31-Dec-20	Confirmed	—
83	F	A	9	31-Dec-20	Confirmed	—
90	F	A	11	31-Dec-20	Confirmed	—

a Date positive indicates the date the RT-qPCR swab returned a positive result for COVID-19. A dash in the SARS-CoV-2 lineage column indicates the sample was not sequenced. Two patients stayed in two different rooms in ward D (e.g., 3 and 17) to cohort COVID-positive or -recovered patients.

### Using contact tracing and epidemiological data to deduce transmission patterns.

**FIG 1:**
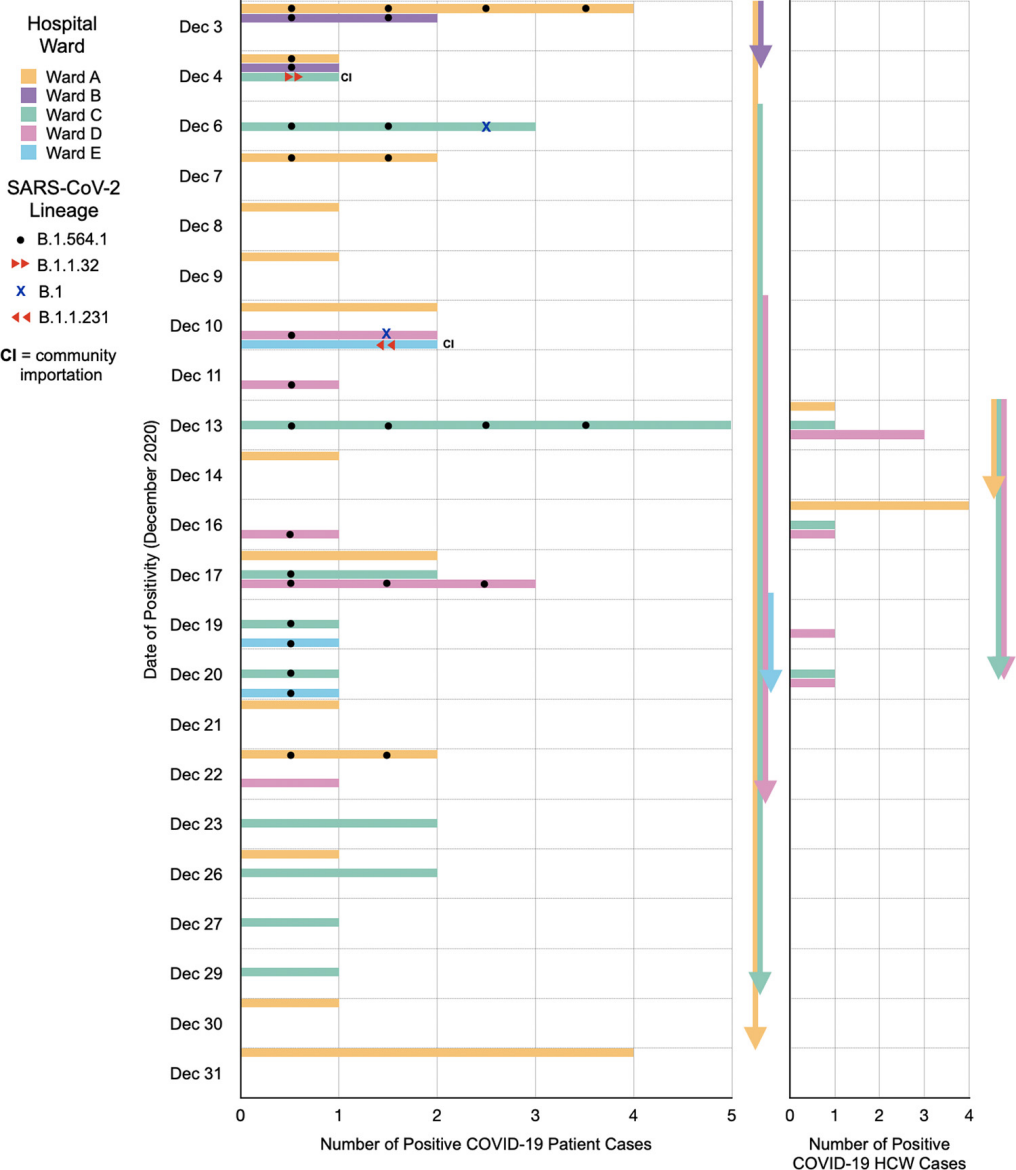
Ward attribution and SARS-CoV-2 lineage variant descriptions of the positive COVID-19 patient and HCW cases. COVID-positive cases that were considered importations from the community into the hospital based on lineage assignment alone are indicated as CI. Arrows depict the duration of time of COVID-positive patient and/or HCW cases within a ward. All 58 patients and 14/48 HCWs had known COVID-positive test dates.

Across all 58 COVID-positive patient cases, most of the cases were attributed to wards A (*n* = 23, 39.7%) and C (*n* = 19, 32.8%). For 14 HCWs, their infection could be attributed to a particular ward, where 35.7% (*n* = 5), 21.4% (*n* = 3), and 42.9% (*n* = 6) of COVID-positive HCWs were attributed to wards A, C, and D, respectively (Fig. 1). Wards A, C, and D had the longest outbreak periods (Fig. 1; Table 2). In ward A, the epidemiologic data are suggestive of COVID-19 transmission from patients to HCWs and back to patients (Fig. 1). However, it is difficult to confirm given the lack of certainty of ward attribution of COVID-positive HCWs. Employee Health Services did not provide ward assignments for each HCW to protect their confidentiality and privacy. In addition, HCWs often work across multiple wards. A description of the outbreak in each ward is summarized in Table 2.

**TABLE 2:** Summary of the outbreak in each ward with SARS-CoV-2 lineage descriptions

Ward	Outbreak start (mo, day, yr)	Outbreak end (mo, day, yr)	Source of outbreak	Total no. of COVID-19-positive patients	No. of SARS-CoV-2-sequenced samples assigned to a lineage	SARS-CoV-2 lineage(s)
A (medicine)	Dec. 3, 2020	Jan. 5, 2021	Patient exposed to a COVID-19-positive HCW	23	9	B.1.564.1 (*n* = 9/9)
B (cardiology and medicine)	Dec. 4, 2020	Dec. 18, 2020	Three patients with exposure to the same COVID-19-positive HCW from ward A	3	3	B.1.564.1 (*n* = 3/3)
C (alternate level of care, mostly medicine)	Dec. 6, 2020	Jan. 12, 2021	Four patients exposed to two COVID-19-positive patients in ward A during their infectious period	19	11	B.1.564.1 (*n* = 9/11), B.1 (*n* = 1/11), B.1.1.32 (*n* = 1/11)
D (surgery)	Dec. 11, 2020	Jan. 1, 2021	Began with two cases, one of them with no direct epidemiological link to the other outbreak cases	9	7	B.1.564.1 (*n* = 6/7), B.1 (*n* = 1/7)
E (rehabilitation)	Dec. 11, 2020	Jan. 4, 2021	All patients exposed to a positive HCW	4	3	B.1.564.1 (*n* = 2/3), B.1.1.231 (*n* = 1/3)

### Prevalence of SARS-CoV-2 lineages.

To investigate the genomic epidemiology of the outbreak, we sequenced 33/58 (56.9%) patient samples and 29/48 (60.4%) HCW samples. The SARS-CoV-2 B.1.564.1 lineage was the most prevalent across all COVID-positive patient and HCW samples that were sequenced (Table 3). Within the patient population, the SARS-CoV-2 B.1.564.1 lineage was the most prevalent (87.8%, *n* = 29), followed by SARS-CoV-2 B.1 (6.1%, *n* = 2) (Table 3). One patient sample was sequenced but failed to be assigned to a defined lineage at the time of analysis. Across the affected HCW population, the SARS-CoV-2 B.1.564.1 lineage was also the most prevalent (82.8%, *n* = 24) (Table 3). As such, from a contract tracing perspective, the outbreak is defined as all cases included in this study, except for those described as nonnosocomial, whereas from a genomic epidemiology perspective, the outbreak is defined as all SARS-CoV-2 B.1.564.1 cases since it is the most prevalent and there is support from contact tracing.

**TABLE 3:** SARS-CoV-2 lineages identified in patient and HCW samples^*a*^

SARS-CoV-2 lineage	Total no. of sequenced samples (*n* = 62)	No. of patient samples (*n* = 33)	No. of HCW samples (*n* = 29)
B.1.564.1	55	29	24
B.1	5	2	3
B.1.1.231	2	1	1
B.1.36.26	1		
B.1.2	1		1
B.1.36.18	1		
B.1.1.64	1		
B.1.1.32	1	1	
B.1.1.39	1		

a The number of Canadian samples of each SARS-CoV-2 lineage was extracted from GISAID on 5 April 2021 for B.1.564.1 and 25 January 2021 for all other lineages. Sequencing was performed on 33 samples from patients and 29 samples from HCWs.

### Comparing contact tracing and genomic epidemiology to elucidate nosocomial and community transmission dynamics.

**FIG 2:**
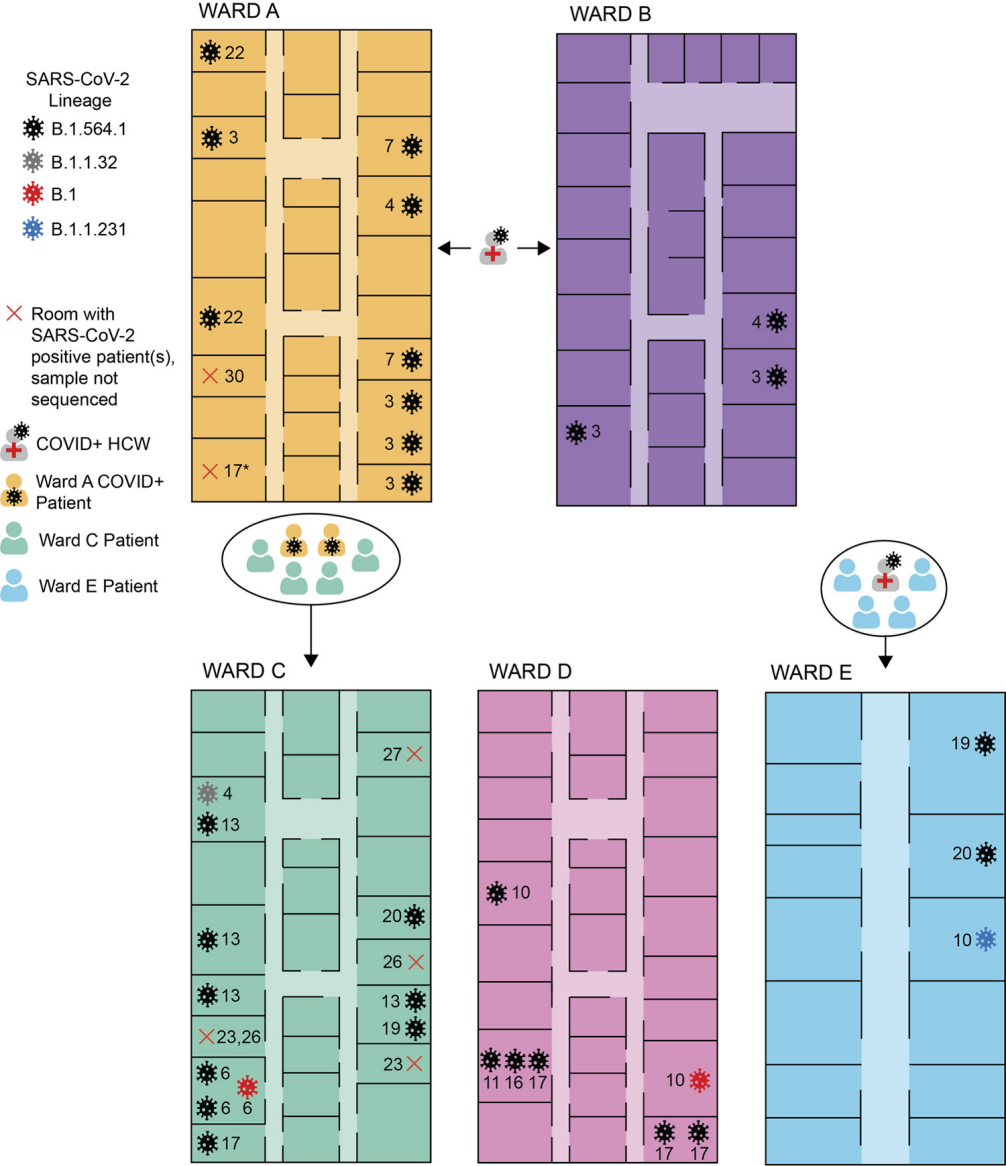
Visual illustration of the COVID-positive patient cases in wards A to E. The number beside each of the viruses indicates the date in December 2020 that the patient sample returned positive for COVID-19 (e.g., 3 indicates 3 December 2020). Diagrams of each of the wards are not to scale. The asterisk indicates a COVID-19-positive case that was confirmed on 17 December 2020 but is either attributed to room 17 or room 7, which already had a sequenced COVID-19-positive case (7 December 2020). Information on the outbreak based on classical epidemiology (e.g., known contact events) is described by the patient and health care worker (HCW) symbols. A COVID-positive physiotherapist was known to move between wards A and B, but directionality of transmission is unclear. Four ward C patients were in contact with two COVID-positive (COVID+) ward A patients. There were no known exposure events in ward D. All 4 patients in ward E were exposed to one COVID-positive HCW, but only 3/4 patients had their COVID sample sequenced, which is depicted in the diagram.

A summary of the genomic epidemiology of the outbreak across five wards is shown in Fig. 1. Most of the sequenced samples were from COVID-positive patients in ward C (32.3%, *n* = 11), followed by 26.5% (*n* = 9) in ward A, 20.6% (*n* = 7) in ward D, 11.8% (*n* = 4) in ward B, and 8.8% (*n* = 3) in ward E (Fig. 2; Table 2). The following paragraphs compare the use of contact tracing and genomic epidemiology to study the outbreak across the five wards, and these findings are visually represented in Fig. 2 and summarized in Table 4.

**TABLE 4:** Comparing contact tracing and genomic epidemiology in studying the outbreak^*a*^

Ward or no. of events or cases	Contact tracing	Genomic epidemiology
Ward A	No. of cases: 23	No. of cases sequenced: 9 All B.1.564.1 lineage
	First case: 3 December 2020 Last case: 31 December 2020	First case: 3 December 2020 Last case: Earliest 22 December 2020
	Physiotherapist moving between wards A and B (directionality unknown) Source: unclear if it is ward B	
Ward B	No. of cases: 3	No. of cases sequenced: 3 All B.1.564.1 lineage
	First case: 3 December 2020 Last case: 4 December 2020	
	Physiotherapist moving between wards A and B (directionality unknown) Source: unclear if it is ward A	
Ward C	No. of cases: 19	No. of cases sequenced: 11 B.1.564.1 (*n* = 7) likely spread from ward A to ward C B.1 (*n* = 1) and B.1.1.32 (*n* = 1) are likely community importations
	First case: 4 December 2020 Last case: 29 December 2020	First case: 6 December 2020 (date of first B.1.564.1 case) Last case: Earliest, 20 December 2020 (date of last B.1.564.1 case)
	No community importations	At least 2 community importations
	Four patients in ward C were exposed to two COVID-positive patients in ward A Source: ward A	
Ward D	No. of cases: 9 One of the two earliest cases had no direct epidemiological link to other cases	No. of cases sequenced: 8 B.1.564.1 lineage (*n* = 6) B.1 lineage (*n* = 1) Since the two earliest cases were B.1.564.1 and B.1, it suggests that B.1 sample is the one that has no direct epidemiological link to other cases.
	First case: 10 December 2020 Last case: 22 December 2020	First case: 10 December 2020 Last case: Earliest, 17 December 2020 (last B.1.564.1 case)
	No community importation	At least 1 community importation
	Source: unclear	
Ward E	No. of cases: 4 All patients exposed to one COVID positive HCW	No. of cases sequenced: 3 B.1.564.1 lineage (*n* = 2) B.1.231 lineage (*n* = 1) Case with B.1.231 lineage is likely due to community importation or exposure to different patient or HCW
	First case: 10 December 2020 Last case: 20 December 2020	First case: 19 December 2020 (first B.1.564.1 case) Last case: 20 December 2020 (last B.1.564.1 case)
	No community importations	At least 1 community importation
	Source: COVID-positive HCW	
No. of cases in the outbreak	All cases (*n* = 106)	At least all sequenced B.1.564.1 lineage (*n* = 55)
Index case	Unknown	
No. of importations into hospital	At least 1	At least 9 (1 from each lineage in Table 3)
No. of within-room transmission events	15	5
No. of neighboring room-to-room transmission events	Ward A: 5 Ward B: 1 Ward C: 7 Ward D: 1 Ward E: 2	Ward A: 3 Ward B: 1 Ward C: 2 Ward D: 0 Ward E: 1

a Concordances and differences between contract tracing and genomic epidemiology are indicated by merged and split cells, respectively. One within-room transmission event in contact tracing is counted as at least two COVID-positive patients in one room with positive tests within a 20-day transmission range. We observed whether genomic epidemiology supports these contact tracing room transmission events by establishing the number of events where lineage assignment was B.1.564.1. Similarly, we counted neighboring room-to-room transmission contact tracing events as adjacent rooms with at least two COVID-positive patients within a 20-day transmission range. Last, we identified support for neighboring room-to-room transmission contact tracing events if the lineage for these sequences was B.1.564.1. All of the contact tracing transmission events that could not be supported by genomics was attributed to not sequencing all patient samples.

In ward A (medicine ward), the first patient sample returned a COVID-positive result on 3 December 2020, and the last sample to return COVID positive was on 31 December 2020. During this period, 23 patient cases were identified, 9 of which were sequenced and typed as SARS-CoV-2 B.1.564.1. Use of genomics supports SARS-CoV-2 B.1.564.1 as the outbreak lineage; however, the majority of the patient cases in ward A were not sequenced. While the last COVID-positive patient sample to return positive was on 31 December, the last SARS-CoV-2 B.1.564.1 sequenced case was on 22 December 2020. Therefore, it is unclear whether the outbreak in this ward ended on 22 December 2020 or 31 December 2020.

In ward B (cardiology and medicine ward), all positive COVID-19 results were received between 3 December 2020 and 4 December 2020. Three patient cases were detected, and their samples were sequenced and assigned to SARS-CoV-2 B.1.564.1. Genomic sequencing supported these three patient cases to be a part of the outbreak since they were all classified as lineage B.1.564.1.

A physiotherapist that moved between wards A and B was thought to be the common link between the wards, but the directionality of transmission is unknown. It is unclear whether the first patient case was from ward A or B, as six samples assigned to SARS-CoV-2 B.1.564.1 were all identified on the first date of the outbreak, 3 December 2020.

In ward C (alternate level of care, mostly medicine ward), the first COVID-positive result was identified on 4 December 2020, and the last patient sample to return COVID positive was on 29 December 2020. In total, there were 19 patient cases, 11 of which were sequenced. The earliest sample was assigned to lineage B.1.1.32. Following this case, on 6 December 2020, there were two samples assigned to lineage B.1.564.1 and one to lineage B.1. Using contact tracing, the start of the outbreak would have been 4 December 2020, whereas using genomics, the start of the outbreak would be considered 6 December 2020. The remainder of the COVID-positive samples (63.6%, *n* = 7) were assigned to B.1.564.1. Four patients in ward C were exposed to two COVID-positive patients in ward A, which is the best explanation we have for the spread of SARS-CoV-2 B.1.564.1 from ward A to ward C. The first SARS-CoV-2 B.1.564.1 cases from wards A and C were on 3 December 2020 and 6 December 2020, respectively, which also supports the transmission from wards A to C. Using only contact tracing, we would have considered all of the cases to be part of the outbreak, but the two patient samples assigned to SARS-CoV-2 B.1 and B.1.1.32 were likely community importations since they were not classified as B.1.564.1. The SARS-CoV-2 B.1 and B.1.1.32 cases were not suspected to be community importations based on contact tracing, and it is unclear whether these were the patients who interacted with ward A COVID-positive patients or patients from another ward.

In ward D (surgery ward), nine patient cases were identified between 10 December 2020 and 22 December 2020. All patient samples sequenced were assigned to lineages B.1.564.1 (85.7%, *n* = 6) and B.1 (14.3%, *n* = 1). The first two COVID-positive samples from 10 December were assigned to lineages B.1.564.1 and B.1. Based on contact tracing, at least one of these first two cases had no known contact with any other outbreak case. Only due to lineage assignments are we able to suspect that the B.1 case could have been an independent community importation since it differs from the outbreak lineage, B.1.564.1. The source of transmission to ward D remains unclear but could be explained by a staff member who acquired infection in the community or from another ward, or a patient visitor who acquired infection in the community.

In ward E (rehabilitation ward), there were four patients detected, and three of these samples were sequenced. The cases were identified between 10 December and 20 December. All four patients were exposed to one positive HCW. With contact tracing only, we would have assumed that all four patients were part of the outbreak, as they had the same exposure. While all patients had similar known exposure, two patient samples were assigned to SARS-CoV-2 B.1.564.1, and one patient sample was assigned to SARS-CoV-2 B.1.231. Only through genomics were we able to identify two different lineages, which suggests that the less common lineage (SARS-CoV-2 B.1.231) was potentially introduced by a community exposure or possibly by another infected staff member. In addition, the first case of SARS-CoV-2 B.1.564.1 returned positive on 19 December 2020, suggesting that the outbreak may not have begun in this ward on 10 December 2020. However, there was one COVID-positive sample from 10 December 2020 that was not sequenced, so the start date is inconclusive.

Using contact tracing, we would estimate that there was at least one importation into the hospital. There were multiple first cases across wards A and B; thus, there could have been one or more via importation. Additionally, there were a few cases where the patient was COVID positive at or near the date of admission; therefore, these cases could have plausibly been unlinked to the outbreak. With genomics, we identified 9 different lineages; therefore, we estimate that there were at least 9 importations into the hospital (Table 3). There may have been multiple importations of the same lineage. In other words, we were able to exclude the linkage of at least 9 cases using lineage assignments, but with contact tracing, it is less clear since sometimes positive COVID tests were collected on or near the date of admission. Based on genomic epidemiology, it is likely that not all of the SARS-CoV-2 B.1.564.1 cases were linked. We identified evidence for the spread of SARS-CoV-2 short- and long-range (i.e., person-to-person and room-to-room, respectively) transmissions. We estimated 15 within-room (i.e., person-to-person) transmission events by only using contact tracing and counting the frequency of at least two COVID-positive patients in the same room, with a transmission window of 20 days. With genomics, lineage assignments were able to support 33% (*n* = 5) of these within-room transmission events in wards A, C, and D. There was contact tracing evidence for 16 room-to-room transmission events within a 10- to 20-day infectious period across all wards. Lineage assignments supported 44% (*n* = 7) of long-range transmission events in four wards. Regardless of using contact tracing or genomic epidemiology, emphasis should be placed on excluding linkage events rather than including or supporting linkage events.

### Using phylogenetics and clustering to understand the Canadian context.

Phylogenetic trees were generated to explore this SARS-CoV-2 B.1.564.1 outbreak in an evolutionary context. We only included sequences from the most prevalent SARS-CoV-2 lineage to meaningfully interpret the genomic context.

**FIG 3:**
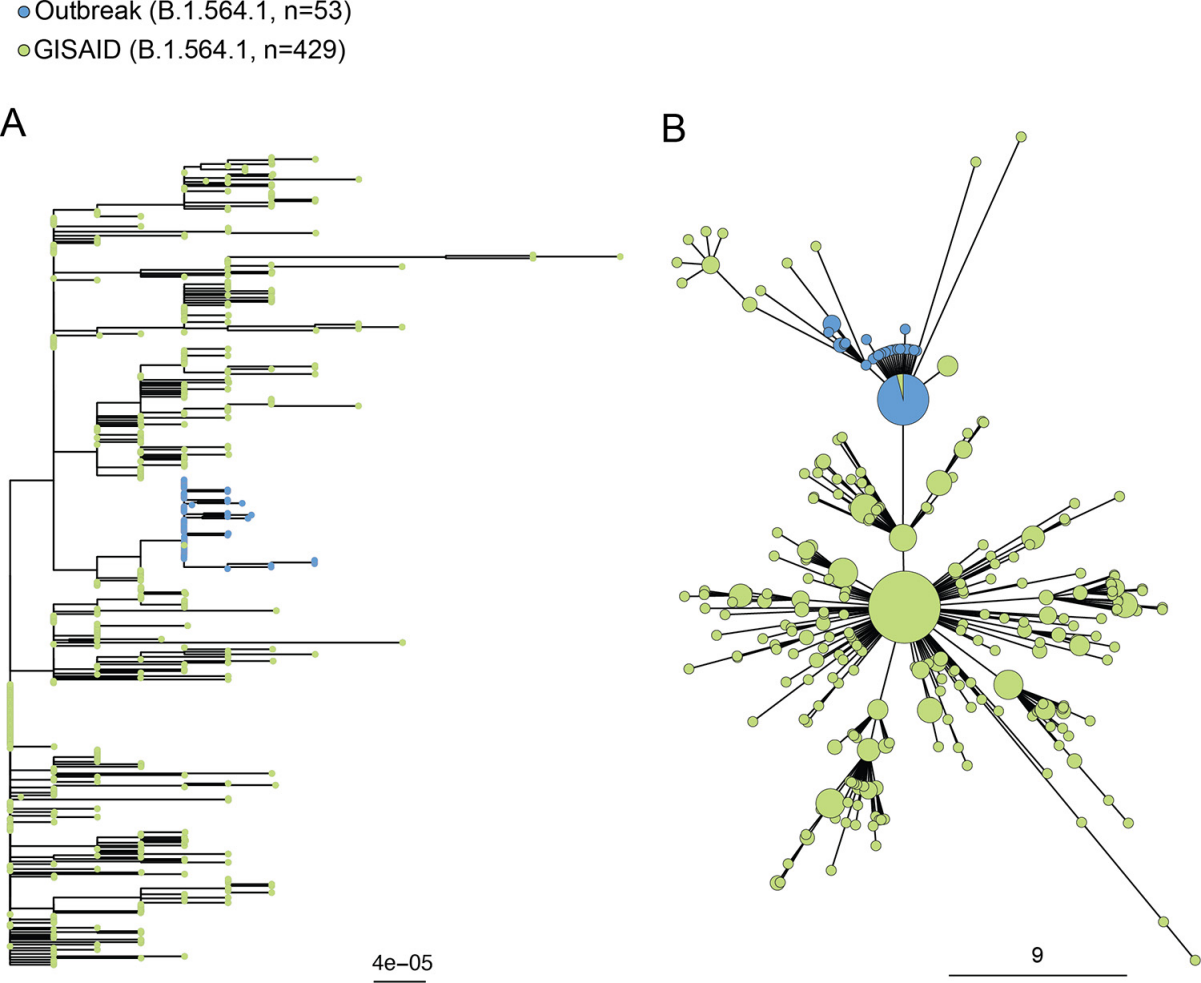
Phylogenetic tree (A) and minimum spanning tree (B) with the scales in nucleotide distances of outbreak samples and Canadian SARS-CoV-2 B.1.564.1 sequences from GISAID. Sequences from GISAID are shown in green, whereas sequences from the outbreak are shown in blue. The size of the circle in the minimum spanning tree is proportional to the number of sequences.

**FIG 4:**
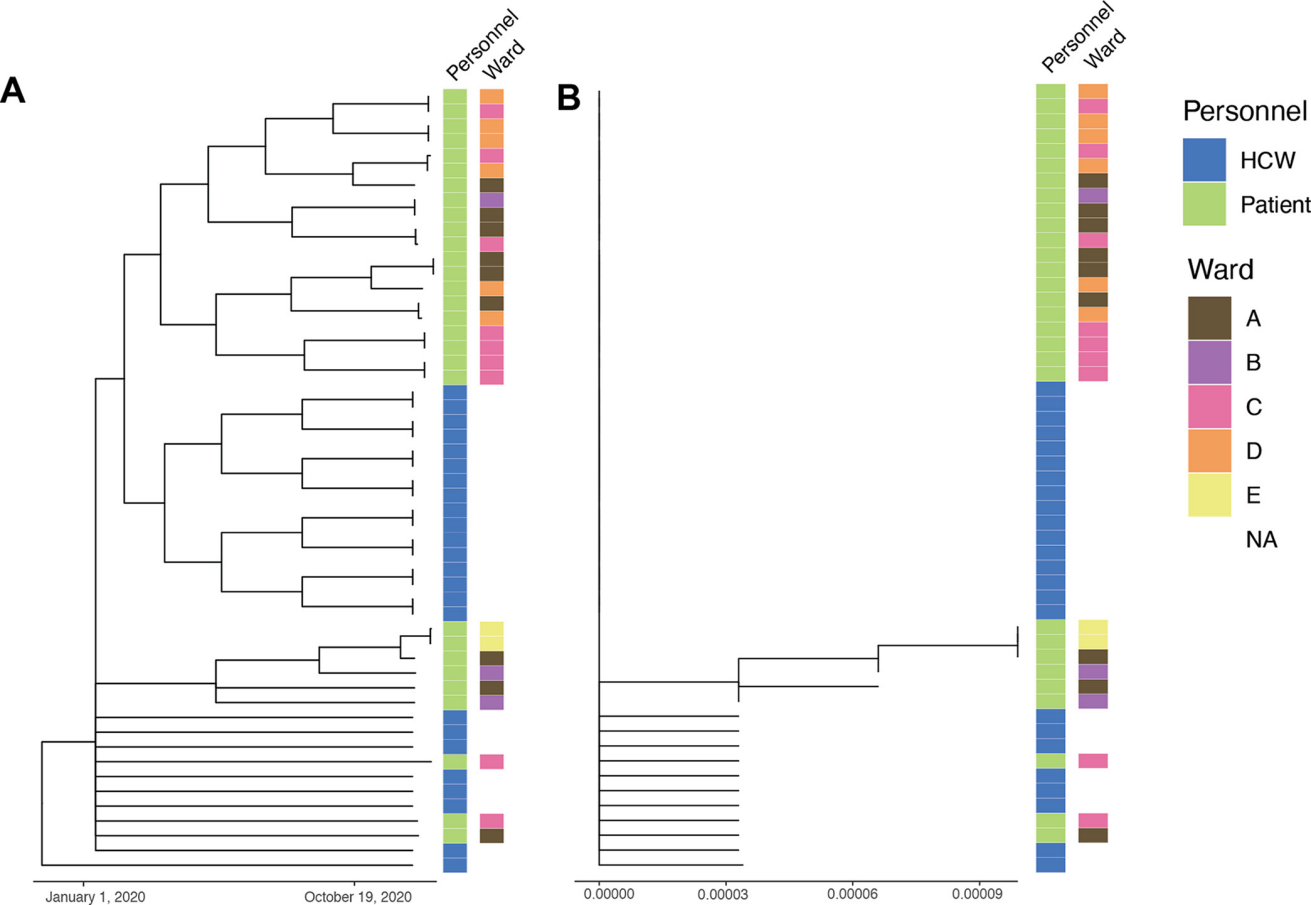
Time tree (A) and divergence tree (B) generated using only SARS-CoV-2 B.1.564.1 outbreak samples annotated with personnel and ward. Branch length corresponds to time and the number of mutations for the time and divergence tree, respectively. Sample source (e.g., health care workers [HCWs] or patients) and ward information from patients only are annotated beside each tree.

We generated a phylogenetic and minimum spanning tree with the available Canadian SARS-CoV-2 B.1.564.1 sequences from GISAID and our outbreak sequences (Fig. 3). The sequences from GISAID included sequences that originated from the same province where the outbreak occurred, Ontario (*n* = 424), in addition to Quebec (*n* = 4) and British Colombia (*n* = 1). The outbreak sequences were grouped together and in the middle of the phylogenetic tree with the Canadian SARS-CoV-2 B.1.564.1 sequences (Fig. 3A). In the minimum spanning tree, the outbreak sequences are clustered together and isolated from most of the other Canadian GISAID SARS-CoV-2 B.1.564.1 sequences, suggesting that the outbreak sequences are most similar to each other and less similar to the other Canadian GISAID SARS-CoV-2 B.1.564.1 (Fig. 3B). In both trees, there is one GISAID SARS-CoV-2 B.1.564.1 sequence that clusters within the outbreak sequences (Fig. 3). This sequence (GISAID ID: EPI_ISL_792075) was collected from an outpatient in Brampton, Ontario, on 24 November 2020. Brampton is 70 km from Hamilton, is connected by major highways, and has many social and economic connections. To further investigate the relationship between the outbreak sequences, we generated a time and divergence tree with patient and HCW characteristics (e.g., date of positive COVID-19 result and patient location) (Fig. 4). The time and divergence trees had the same clustering, but we could not identify or resolve any relationships within the outbreak samples relative to the characteristics that were plotted.

## DISCUSSION

We used traditional contact tracing and next-generation sequencing methods to understand a SARS-CoV-2 B.1.564.1 outbreak that occurred across five wards. The five wards where the outbreak occurred were initially constructed between the 1930s and 1960s and had renovations in the 2010s. The units contain mostly multibed rooms, and the outbreak transmission pattern aligns with short-range transmission typically observed with SARS-CoV-2 (7–9) to roommates, with possible patchy spread across the unit from mobile HCWs working during their infectious period, as opposed to rapid long-distance transmission such as measles and varicella outbreaks (10–13). Sporadic clusters of nosocomial COVID-19 infections have also been observed, even in highly vaccinated populations and with N95 usage (14) compared to our study setting with low vaccination and surgical mask usage. The COVID infection rate in the hospital was 275× higher than in the community, exemplifying the magnitude of the outbreak.

There have been many research articles performing SARS-CoV-2 outbreak analyses that use genomic and epidemiologic data (2–6) but only a few sequenced COVID-positive samples from HCWs (3, 4, 15–19). Many of these outbreak analyses vary largely in the contact tracing information that they collected, as well as the sequencing breadth (i.e., number of outbreak samples sequenced) and depth (i.e., quality of sequencing). The data collected dictated what each study was able to understand about their outbreak. For example, one study was able to use whole-genome sequencing to identify their index case (5). While another study was not able to identify their index case, they were able to identify some significant transmission events between patients and HCWs (15). Similarly, in our work, we were not able to identify the index case, but we did find evidence of transmission between patients and HCWs through the spread of the SARS-CoV-2 B.1.564.1 lineage across five wards (Fig. 2), as well as evidence of specific instances of patient-to-patient and HCW-to-HCW transmission. This suggests that the outbreak was not just reflective of a rise in community SARS-CoV-2 with repeated hospital introduction of the virus. The source of the outbreak would have been falsely assumed, but instead, evidence of spread of a predominant lineage was suspected based on basic epidemiological observation. We also saw evidence of a few instances of community importations, for example, the identification of SARS-CoV-2 lineages B.1.1.32, B.1.1.231, and B.1 in three wards. The one external sequence from an outpatient in Brampton, Ontario, collected prior to the outbreak clustering with outbreak sequences suggests that this outbreak may have begun from a community or neighboring hospital importation.

Last, one common aim of an outbreak analysis is to determine whether there was involvement of a SARS-CoV-2 variant of concern (VOC), as they may have increased transmissibility (20). In our outbreak, our results show that there was no involvement of a new SARS-CoV-2 VOC, particularly the Alpha variant, which was introduced to Ontario around the same time.

Altogether, similar to other studies (14, 21, 22), we show the value of combining contact tracing and genomic epidemiology to understand different aspects of the outbreak that could not be possible with only either contact tracing or genomic epidemiology alone. While we were able to learn about the genomic epidemiology, transmission, and geographical context of this outbreak, there were a few limitations of this study. Due to confidentiality and privacy, we were unable to collect more detailed HCW information. Some staff may have also worked on multiple units, adding to the complexity of ward assignment. We were unable to collect ward attribution and the date of the positive COVID-19 result to the SARS-CoV-2 lineage for the majority of HCWs. If we had ward attribution, date of positive COVID-19 results, and SARS-CoV-2 lineage for each HCW, we would have a better understanding of the patient-to-HCW (or vice versa) transmission. The value of HCW data can be exemplified by a study that showed that most importation events were linked to HCWs and that transmission was limited between HCWs (21). Our study also lacks community contact tracing, which was shown to be important for clarifying nosocomial transmission of SARS-CoV-2 in Singapore (23). In addition, we were unable to recover (e.g., possibly an inability to find or not stored) some (44/106, 41.5%) of the specimens for sequencing retrospectively; if we sequenced all patient and HCW COVID-positive cases from every room, we would have been able to trace the outbreak with greater resolution and likely resolve transmission chains more than the nine cases we were able to reject using lineage assignments only. Since our study was retrospective, we were not able to implement any outbreak control measures. However, previous uses of genomic sequencing have resulted in structured and efficient public health measures to control local transmission in the Netherlands and Australia (24). In addition, prospective methodologies have been developed to combine epidemiological and genome sequence data to provide a rapid assessment of nosocomial infections that would be useful for infection prevention and control (25).

Our results are an interpretation of the available data with the understanding that missing clinical, employee health, and sequencing information did lead to a fragmented investigation of this outbreak and its transmission. At the time, there was inconsistent genomic surveillance of SARS-CoV-2 in the community, making it difficult to place transmission within a broader context. Notably, our local community now has ongoing weekly genomic surveillance of SARS-CoV-2 variants and reporting to Public Health Ontario, plus new variant sequencing capacity within our hospital network, which allows rapid resolution of hospital outbreaks and community transmission.

In summary, sequencing of this multiward outbreak showed that most cases were indeed due to in-hospital transmission events rather than new introduction of different lineages during the outbreak period. The chain of transmission could be explained by transmission through close contact, driven by patient-to-patient transmission in multibed rooms, but also evidence of staff-to-staff transmission and transmission from staff to patients and vice versa.

## MATERIALS AND METHODS

### Setting.

On 3 December 2020, during the second wave of the coronavirus disease 2019 (COVID-19) pandemic in Ontario, a COVID-19 outbreak started in an acute medicine ward in a 379-bed (across 18 wards) tertiary acute care and regional cancer center hospital in Ontario, Canada. Over a 1-month period, the outbreak spread over five different wards (medicine, cardiology and medicine, alternate level of care, surgery, and rehabilitation) within the hospital (see Table S1 in the supplemental material). The wards are spread throughout the hospital, with wards A and B on the same floor. The number of rooms in each ward ranges from 12 to 16, with each room occupying a range of 1 to 4 beds. The average number of patients and health care workers (HCWs) in each ward ranged from 10 to 28 and 2 to 5, respectively. This outbreak preceded transmission of the SARS-CoV-2 B.1.1.7 (Alpha) variant of concern (VOC) in the city and well before the first case of B.1.617.2 (Delta VOC).

### Case identification and contact tracing definitions.

Since March 2020, both patients and HCWs have been screened daily for symptoms of COVID-19. If the patient symptom screen is positive, e.g., symptoms developed, patients were isolated and tested with a nasopharyngeal RT-qPCR test for SARS-CoV-2. HCWs were required to self-report any symptoms and complete a screening form upon arrival at the hospital. Confirmed COVID-19 cases were defined based on a positive RT-qPCR swab test. Probable and confirmed hospital-acquired infections are defined in Table S2. Generally, if a patient tested COVID positive, they were moved out of the room to either a COVID-dedicated room or to be cohorted with other COVID-positive or -recovered patients. Once a positive hospital-acquired COVID-19 case (patient and/or HCW) was confirmed, Public Health (PH) was notified, and a thorough contact tracing was initiated for 48 h before the onset of the symptoms. An outbreak was declared when there were at least two patients and/or HCW hospital-acquired COVID-19 cases.

### Outbreak control measures.

Since March 2020, universal masking of all HCWs and ambulatory patients, physical distancing and room capacity restrictions, and universal symptom-based COVID screening daily of all patients and HCWs were implemented. To follow provincial infection prevention and control guidelines, when an outbreak is declared, additional measures were put in place, which are described in Table S3 (26, 27).

### Data collection.

Patient data, including demographics, date of detection, unit attribution, and mortality outcomes, were collected retrospectively from the infection control database. Employee date of detection and unit attribution were obtained from the Environmental Health & Safety (EHS) database. Contact tracing information was extracted from daily screening forms for patients and HCWs.

### PCR testing for SARS-CoV-2 and sequencing methods.

Samples were initially identified as being positive for COVID-19 through the Hamilton Regional Laboratory Medicine Program using clinically validated laboratory-developed test (LDT). Depending on the ordering requirements, samples were tested via the BD Max or Hamilton Star. For samples tested via the BD Max, approximately 250 μL of sample was added to the BD Max ExK TNA-2 sample buffer tube and then placed on the BD Max for integrated extraction and amplification. Amplification was performed using Luna universal probe one-step RT-qPCR master mix (catalog no. E3006; New England Biolabs), primers and probes targeting the SARS-CoV-2 envelope and 5′ untranslated region (UTR), and human sample adequacy marker, RNase P. For samples tested via the Hamilton Star, 250 μL of sample was transferred directly from the original sample collection tube to the sample processing plate. The samples in the processing plate were then subjected to extraction using the Maxwell HT viral TNA kit (catalog no. AX2340; Promega). Following extraction, the Hamilton Star was then used to set up the RT-qPCR using the purified nucleic acid and Luna universal probe one-step RT-qPCR master mix, primers and probes targeting the SARS-CoV-2 envelope and 5′ UTR, and human sample adequacy marker, RNase P. Amplification was performed on a Bio-Rad CFX96 Touch real-time PCR detection system.

Once a positive specimen was identified, the sample was reextracted via the bioMérieux easyMAG to yield purified nucleic acid for downstream sequencing applications. Briefly, 250 μL of sample was added to 2.0 mL of easyMAG lysis buffer. After incubating for 10 min to inactivate, the total volume was transferred to the easyMAG extraction cartridge. Fifty microliters of NucliSens easyMAG magnetic silica was added to the sample and lysis buffer mixture and then mixed thoroughly by pipetting. The samples were then processed using the Generic 2.0.1 protocol, and eluate volume was set to 40 μL.

Prior to sequencing library preparation, samples with a high viral load (PCR cycle threshold [*C_T_*] of less than 16) were diluted 10× in DNase/RNase-free water. Library preparation followed the ARTIC v3 SARS-CoV-2 amplification protocol (as described in https://artic.network/ncov-2019). Library preparation began with a priming reaction consisting of random hexamers, deoxynucleoside triphosphates (dNTPs), and DNase/RNase-free water according to the manufacturer’s specifications (SuperScript IV first-strand synthesis system; Invitrogen, Carlsbad, California). DNA amplification using ARTIC v3 primers (Integrated DNA Technologies, Coralville, IA) was then carried out to amplify viral RNA using Q5 Hot Start HiFi master mix (New England Biolabs, Whitby, Ontario), ARTIC v3 primer pools (Integrated DNA Technologies, Coralville, IA), and DNA/RNase-free water. To perform the PCR, samples were heated to 98°C for 30 s before 35 cycles of 15 s at 98°C and 5 min at 63°C. Samples were then run on a gel to confirm controls did not contain amplicons of the expected band size of approximately 500 bp. ARTIC pools were then combined, followed by magnetic bead purification (Promega, Madison, WI), with the beads added at an amount of 1.25× the PCR product. Samples were washed using 80% ethanol before being eluted in 10 mM Tris-HCl, pH 8.5 (Promega), followed by end repair according to the manufacturer’s specifications (New England Biolabs, Ipswich, MA). The mixture was then incubated in a thermal cycler for 30 min at 20°C followed by 30 min at 65°C before adaptors (New England Biolabs) were ligated to DNA by heating at 15 min at 20°C. Uracil-specific excision reagent (USER) enzyme and CutSmart buffer (New England Biolabs) were then applied to the DNA mixture according to the manufacturer’s specifications before heating at 37°C for 15 min. Magnetic bead purification was performed once again, using beads at an amount of 1.12× the ligated DNA. Illumina i5 and i7 barcodes (Illumina, San Diego, CA) were attached to samples through limited PCR, with denaturation at 98°C for 10 s and annealing at 65°C for 75 s and repeating this process 9 times. Two final bead purifications were performed, first using 0.91× the number of beads compared to DNA and then using 0.16× beads compared to the amount of DNA. Paired-end 150 bp sequencing of the resulting libraries was then performed using an Illumina MiSeq (Illumina) to generate genome sequence consensus sequences based on ~2000× coverage. Sequencing depth for libraries was based on both scientific objectives and turnaround time, barcode and reagent availability, and device availability constraints during the pandemic. These constraints led to a depth of sequencing surpassing what is needed for the scientific objectives to avoid undue delays.

### Genomic epidemiology and phylogenetic analyses.

All sequencing results were analyzed using the SARS-CoV-2 Illumina GeNome Assembly Line (SIGNAL, v1.4.4) software (28), a standardized bioinformatics workflow for short-read SARS-CoV-2 genome sequencing (https://github.com/jaleezyy/covid-19-signal), as well as the NCoV-Tools (v1.8.0) workflow for control and quality assessment (https://github.com/jts/ncov-tools) (28). Within SIGNAL, mutation calling was performed using the FreeBayes (29) (v1.3.2, consensus minimum depth = 10) option. SARS-CoV-2 lineage assignment of the consensus assemblies was performed using the latest versions of the Phylogenetic Assignment of Named Global Outbreak Lineages (PANGOLIN; https://github.com/cov-lineages/pangolin) and NextClade (30) software. For the phylogenetic analyses, 429 SARS-CoV-2 B.1.564.1 sequences from Canada were downloaded from GISAID (31) on 5 April 2021 (covering March to December 2020). A minimum spanning tree that included only the outbreak sequences (SARS-CoV-2 B.1.564.1) and GISAID sequences was generated and visualized using GrapeTree (32). MAFFT (33) (v7.455) was additionally used to align the genome sequences, and FastTree (34) (v2.1.10) was used to build approximate maximum-likelihood phylogenetic trees. To generate the time and divergence trees of only the outbreak sequences, TreeTime (35) (v0.9.3) was used with the “time-marginal” parameter set to true, and any samples with unknown collection dates were assigned December 2020 as a guide. ggtree (36) (v3.13) was used for all tree visualizations.

### Data availability.

All sequencing reads have been deposited in BioProject under accession no. PRJNA689621, and all high-quality consensus genome sequences were deposited in the GISAID EpiCoV database. All submitted and contributed genome sequences and associated metadata are accessible at https://epicov.org/epi3/epi_set/221120yo?main=true.
