# Supplementary material for: SARS-CoV-2 Outbreak Investigation Using Contact Tracing and Whole-Genome Sequencing in an Ontario Tertiary Care Hospital
Source: Microbiol Spectr. 2023 Apr 24;11(3):e01900-22. doi: 10.1128/spectrum.01900-22 (PMC10269621; doi:10.1128/spectrum.01900-22)
Supplement: Supplemental file 1 — Table S1 to S3. Download spectrum.01900-22-s0001.pdf, PDF file, 0.10 MB [file spectrum.01900-22-s0001.pdf]

Supplementary Material

Supplementary Table 1. Characteristics about each ward between December 2020 and January 2021.

Ward	Average number of patients per month	Number of patients assigned to each HCW (day-night shift) per month	Number of rooms with one bed	Number of rooms with two beds	Number of rooms with four beds
Ward A (Medicine)	28	6-7	4	5	5
Ward B (Cardiology and Medicine)	10	3-6	9	3	0
Ward C (Alternate level of Care, mostly Medicine)	23	6-7	4	5	7
Ward D (Surgery)	24	6-9	4	3	6
Ward E (Rehabilitation)	18	4-6	9	3	4

Supplementary Table 2. Probable and confirmed hospital-acquired infection definitions for patients and healthcare workers.

<table>
<tr><th></th><th>Case type</th><th>Case definition</th></tr>
<tr><td rowspan="2">Patient</td><td>Probable hospital-acquired infection</td><td>Symptom onset and a positive test occurring after at least 5 days of admission with no other epidemiological links</td></tr>
<tr><td rowspan="2">Confirmed hospital-acquired infection</td><td>Symptom onset and a positive test after at least 14 days of admission with no other epidemiological links</td></tr>
<tr><td>Healthcare worker</td><td>Positive test and no known community exposures</td></tr>
</table>

Supplementary Table 3. Control measures implemented for patients and healthcare workers (HCWs) after an outbreak has been declared.

	Control measure	Time period
COVID positive or exposed patients	Droplet/contact precautions	14 days from their last exposure
Patients cared for by or had direct contact with COVID positive HCW	Droplet/contact precautions	14 days from their last exposure
All patients (when COVID positive HCW spends >6 hours on unit or there is direct contact with patient/another HCW)	Point prevalence by RT-PCR	Until no further cases are identified on two sequential point prevalence studies
HCW with high risk exposure (e.g., exposed to COVID positive patient without mask)	Home isolation	14 days
HCW with medium risk exposure (e.g., exposed to COVID positive patient without eye protection)	Home isolation	Can return to work after testing negative on day 7 post exposure
HCW with low risk exposure (e.g., exposed to unmasked COVID positive patient)	Daily symptom screening	
Visitors	Only allowed for compassionate ground (e.g., imminently dying patients)	Until outbreak declared over
Hospital	• Additional cleaning<br>• Increase in hand hygiene and personal protective equipment audits<br>• Universal eye protection<br>• Patient masking for HCW interactions	Until outbreak declared over
